# Psychiatric service staff perceptions of implementing a shared decision-making tool: a process evaluation study

**DOI:** 10.1080/17482631.2017.1421352

**Published:** 2018-02-06

**Authors:** Ulla-Karin Schön, Katarina Grim, Lars Wallin, David Rosenberg, Petra Svedberg

**Affiliations:** ^a^ School of Education, Health and Social Studies, Dalarna University, Falun, Sweden; ^b^ Institution for Social Work, Karlstad University, Karlstad, Sweden; ^c^ Department of Social Work, Umeå University, Umeå, Sweden; ^d^ School of Social and Health Sciences, Halmstad University, Halmstad, Sweden

**Keywords:** Shared decision making, psychiatric services, decision support tool, implementation, process evaluation

## Abstract

**Purpose:** Shared decision making, SDM, in psychiatric services, supports users to experience a greater sense of involvement in treatment, self-efficacy, autonomy and reduced coercion. Decision tools adapted to the needs of users have the potential to support SDM and restructure how users and staff work together to arrive at shared decisions.

The aim of this study was to describe and analyse the implementation process of an SDM intervention for users of psychiatric services in Sweden. **Method:** The implementation was studied through a process evaluation utilizing both quantitative and qualitative methods. In designing the process evaluation for the intervention, three evaluation components were emphasized: contextual factors, implementation issues and mechanisms of impact.

**Results:** The study addresses critical implementation issues related to decision-making authority, the perceived decision-making ability of users and the readiness of the service to increase influence and participation. It also emphasizes the importance of facilitation, as well as suggesting contextual adaptations that may be relevant for the local organizations. **Conclusion:** The results indicate that staff perceived the decision support tool as user-friendly and useful in supporting participation in decision-making, and suggest that such concrete supports to participation can be a factor in implementation if adequate attention is paid to organizational contexts and structures.

## Background

Shared decision making, SDM, in psychiatric care, supports users to experience greater empowerment, including a subjective sense of involvement in treatment, self-efficacy, autonomy and reduced coercion (Stovell, Morrison, Panayiotou, & Hutton, 2016). SDM diverges radically from compliance, which is often a primary focus in treatment planning, since it assumes that two experts—the user and the practitioner—must share their respective knowledge, experience and viewpoints and collaboratively agree upon the choice of treatment. Despite these potential benefits, research on SDM and its implementation in practice in psychiatric care is still at a formative stage (Morant, Kaminskiy, & Ramon, 2015; Stovell et al., 2016). Attempts to describe and measure the benefits of SDM in psychiatric care have often been compromised by poor implementation (Morant et al., 2015; Ramon et al., 2017; Slade, 2017; Stovell et al., 2016). In the field of somatic care, SDM has been described as a process of supportive collaboration between users and staff, drawing on user’s preferences and values, and the best available evidence to discuss multiple options and reach a consensus in care (Duncan, Best, & Hagen, 2010; O’Connor et al., 2007). However, when implementing SDM, the results often suggest that the staff are trying to persuade the user to agree with a particular option, rather than offering them the opportunity to discuss several possible options, as prescribed in the SDM process (Land, Parry, & Seymour, 2017).

Studies investigating the implementation of SDM interventions in somatic care have primarily taken place in the U.S.A. and the UK (Elwyn et al., 2012; Kashaf, McGill, & Berger, 2017; Légaré et al., 2010; Woodhouse et al., 2017). While few in number, implementation studies on SDM interventions in Sweden have suggested the importance of expanding the decision-making process beyond single encounters (Hultberg & Rudebeck, 2017) and beyond simply educating the patient in self-care (Herlitz, Munthe, Törner, & Forsander, 2016). Results from somatic care studies suggest that successful implementation of SDM requires continuity of care and that the user is offered concrete opportunities to participate as an equal in the decision-making process (Elwyn, Frosch, & Kobrin, 2015). Basic prerequisites for successful implementation of SDM in psychiatric care settings are considered to consist of the following factors; (a) attending staff have the ability and are willing to include the user in decisions (skills and attitude) (b) the user is willing and has the ability to actively participate in the decisions (c) additional information and decision support is available to facilitate the SDM process (Hamann, Kruse, Schmitz, Kissling, & Pajonk, 2010; O’Connor et al., 2007). Decision tools, adapted to the needs of users, have the potential to restructure how people with mental illness and staff work together to arrive at shared decisions about the next steps in treatment or support (Deegan & Drake, 2006; National Board of Health and Welfare, 2011). As yet, such tools remain a rarity in the field of psychiatry (Slade, 2017). However, initial studies of existing decision support tools designed for psychiatry show promise for increasing user engagement, satisfaction, knowledge and reductions in decisional conflicts (LeBlanc et al., 2015).

SDM has only been implemented to a limited extent in Sweden and other Scandinavian countries (Légaré et al., 2014), despite the fact that SDM is one of the interventions recommended by the National Board of Health and Welfare in the Swedish Guidelines for Psychosocial Interventions for Schizophrenia or Schizophrenia-type conditions (2011/2017). While these recommendations are primarily based on evidence from Anglo-Saxon practice, the development and dissemination of new methods for psychosocial support have rapidly changed the field of community mental health in Sweden. However, the slow process of rolling out many of these interventions, has led to a growing interest in implementation issues that may be specific to Sweden. General barriers for implementing these interventions, which have their origins in Anglo-Saxon countries, include the misfit between the highly sectorized welfare system in Sweden and the integrative services required by many of the evidence-based interventions (Schön & Rosenberg, 2013). The Swedish constitution also limits the ability of the national government to direct policy and services at the local level. As a consequence, there is often a high degree of adaptation of these different interventions at the local level, and these adaptations often result in reduced fidelity to the original evidence-based models. Systematic reviews report that there is no clear picture of what implementation strategies are most effective. In order to increase opportunities for successful implementation, the importance of tailoring implementation to contextual conditions is regularly emphasized (Damschroder et al., 2009). From a clinical perspective, as well as from a wider implementation research horizon, the need for enhancing knowledge on how to translate evidence on SDM into psychiatric practice is therefore an urgent issue.

This article reports finding related to implementation, which are part of a larger research project focused on the development of evidence-based decision support in SDM in psychiatric care in Sweden. In the research project, researchers, users, staff and service designers have worked together, a partnership which has generated knowledge regarding needs for support that can enable users to participate in their care (Grim, Rosenberg, Svedberg, & Schön, 2016) validated instruments for measuring SDM (Rosenberg, Svedberg, & Schön, 2015; Schön, Svedberg, & Rosenberg, 2015), as well as contributing to the development and validation of a digital interactive decision support tool, *DST*, for users in psychiatric care (Grim, Rosenberg, Svedberg, & Schön, 2017). The *DST* is based on the theoretical framework of SDM (Elwyn et al., 2012) and was adapted to the specific needs and preferences of users with mental health problems in a Swedish context (Grim et al., 2016). This article will therefore focus on the process of implementing the DST in six different psychiatric units in Sweden. The aim of the process evaluation was to explore possible mechanisms of implementation in the context of existing psychiatric services, understand the functioning of the integrated SDM process and identify contextual barriers and facilitators in the various units. We hoped that the findings could help us in understanding and interpreting the outcomes of the effect evaluation of this implementation, as well as providing information regarding the delivery and implementation of the intervention in psychiatric services in the future.

## Aim

The overarching purpose of this study was to describe and analyse the implementation process of a shared decision-making intervention for users with mental illness in Sweden. More specifically, the process evaluation attempted to (1) evaluate whether the implementation program was conducted as planned or if adaptations were made due to the context; (2) understand the barriers and facilitators of the multifaceted implementation program in psychiatric care settings; (3) gain insight into the satisfaction and experiences of staff working with the DST.

## Study design

The study includes three components which will be described below, including the actual clinical intervention which was tested, the implementation program that was developed and the process evaluation that focused on the implementation of the intervention. The participants, however, were common to all aspects of the study.

### Participants

The study was conducted in six units across three different counties in Sweden, all offering psychiatric services to people with severe mental illness. Two of these six units were municipal social services offering residential support services and case management, and four were outpatient psychiatric services offering medical treatment, therapy and home visits. Four of the participating units were recruited from urban areas with about 200 000 inhabitants, and two units were recruited from rural areas with 14 000 to 40 000 inhabitants. Half of these units were located in areas with a socially vulnerable population and a high proportion of foreign born residents. All staff who provided psychiatric care within these units were eligible to participate in the intervention and were therefore included in the data collection. In total, 95 staff participated in the study and are described below in Table I.

**Table I.** Staff characteristics (n = 95).

		Baseline N (%)
Sex^1^	Male	25 (27.1)
	Female	67 (72.9)
Age mean (range)		45 (24–65)
Occupation	Care worker/community support worker	33 (34.7)
	Nurse	19 (20.0)
	Social worker	11 (11.6)
	Occupational Therapist	1 (1.1)
	Psychologist	7 (7.4)
	Case manager	11 (11.6)
	Psychiatrist	6 (6.3)
	Other^2^	7 (7.4)
Years in profession mean (range)		14 (1–40)

### The clinical intervention

The clinical intervention consists of the SDM intervention, in this case an interactive digital decision tool, *DST*, which users and staff were to work with together in the care planning process. The DST features separate, overlapping, web-based interfaces for user and provider, with full transparency between them. The development process and usability evaluation of the *DST* has been described in greater detail elsewhere (Grim et al., 2017). The purpose of using the *DST* is to increase user participation influencing the care planning process, as well as offering staff access to the user’s perceptions of their own needs, knowledge which can facilitate the decision-making process. The *DST* consist of six sections; introduction to the SDM process, presentation of the decision to be made, alternatives to be considered, the details and documentation of the decision itself, and a summary and feedback/follow up, all available online so that the user and the staff can communicate interactively in writing when they have time to log in to the tool. This allows for users to participate at their own pace in settings of their own choice. Each section contains a number of questions (2–5) with some presented as multiple choice and some as open. The questions throughout the tool are formulated in order to support the user in eliciting, verbalizing and communicating the issues and questions that matter most to them, and in legitimizing their values and self-efficacy in the formation of services. Users can easily and securely describe their needs and preferences in the *DST*, the staff can respond with information based on user needs and can suggest possible options for meeting these needs. This allows staff to provide guidance and structure for the user, so that they can take advantage of available information regarding treatment alternatives and make a more knowledgeable decision regarding their care. The *DST* also provides documentation of decisions that the user and staff can go back to and read, and information on how and when decisions will be followed up. Contact information is offered by providers so that if questions or concerns regarding the decisions come up after the meeting, follow-up is available and encouraged. Listening icons with voice files adjacent to descriptive texts are provided and printer icons allow for printable PDF pages. These functions offer individualized formats for sharing information with respect to varied preferences, cognitive challenges, computer literacy and computer access. For providers, “example-phrases” which may help to elicit user input, are provided in the different sections.

### The implementation program

The implementation program describes *how* the clinical intervention is planned to be implemented in the psychiatric services (Table II). The implementation program includes the following primary components (I) the introductory meeting, (II) educating local facilitators, (III) educating staff, (IV) working with *DST*, (V) continuous facilitation, (VI) two follow-up seminars.

**Table II.** Description of the implementation program.

Implementation program components	Content	Time
Introductory meeting	- Introducing service managers and staff to the project - Introduction to the concept of SDM - Presentation of facilitators and their role	Baseline
Educating local facilitators	- Written materials, a film and brief introduction to SDM and to their role in supporting the use of DST among all staff in the services. - One day staff training.	Baseline
Educating staff (1 day)	- Seminar on the theoretical concepts and actual knowledge regarding Recovery orientation in practice and SDM. - Introduction of the DST - Practicing SDM with the DST - Discussion on contextual barriers and facilitators	3 weeks
Working with DST	- Users and staff work together with the DST during a period of 6 months.	1–6 months
Continuous facilitation	- Local facilitators engage and support staff in the implementation of the DST in practice	1–6 months
Follow-up seminar 1 (one half day)	- Dialogue with staff providing opportunities to share experiences of working with implementing SDM and to raise questions and issues regarding the digital decision tool. - Discussion on contextual barriers and facilitators	2 months after education
Follow-up seminar 2 (one half day)	- Dialogue with staff providing opportunities to share experiences of working with implementing SDM and to raise questions and issues regarding the digital decision tool. - Discussion on contextual barriers and facilitators	6 months after education

The implementation program started with an introductory meeting three weeks prior to the first staff education. The purpose of this meeting was to introduce service managers and staff to SDM and to the research project, as well as allowing time for managers to sanction the implementation of the intervention.

Prior to the start of the implementation process, the managers at the participating units were informed regarding the facilitators role in the project and they were asked to choose 1–2 local facilitators at each unit. The local facilitators were assigned to engage and support staff in the implementation of SDM in practice. Reports suggest that facilitators play a key role in the progress and success of the implementation of an intervention, by providing ongoing support to the individuals, teams and organization (der Zijpp et al., 2016; Eriksson et al., 2016; Harvey & Kitson, 2015). To facilitate the implementation of the *DST*, it was expected that the facilitator role would be sanctioned by the head of the service and included as part of the facilitator’s everyday tasks. Facilitator training included written materials, a film and the 1-day staff training. An initial description of the role suggested that one of the facilitators should be a staff member in the service and the other, someone with their own lived experience as a user in psychiatric services, with the specific aim of helping staff to broaden their perspective on decision-making. The facilitators´ role was to offer continuous support to staff in the use of the DST in practice, while themselves receiving supervision from the research team.

The 1-day education was offered to all staff in the units and included an introduction to the theory and evidence base for recovery-oriented practice and SDM (knowledge and attitudes to user participation in care meetings) as well as training in the use of the digital decision tool (skills). The 1-day training was led by two members of the research team, one a PhD student with expertise in SDM and their own experience of mental illness and one, a researcher with expertise in SDM and long experience in teaching recovery and SDM. The digital decision tool was introduced and staff had the opportunity to practice new skills prior to using the tool with users. Prior to the 1-day training, the agenda for the day was pilot-tested and validated with a similar group of staff. This resulted in minor changes regarding instructions for skills exercises and a further development of the digital decision tool, which included adding audio recordings as an alternative to written instructions for the users. Following the education, two follow-up seminars were held with all participating staff to provide opportunities to share their experiences of working with and implementing SDM and to raise questions and issues regarding the digital decision tool.

### The process evaluation

For this process evaluation study, an explorative design was applied using both quantitative and qualitative methods. In designing the process evaluation of the intervention, we developed a blueprint guided by Moore et al. (2015) which included three evaluation components: contextual factors, implementation issues and mechanisms of impact (Table III).

**Table III.** Blueprint of *components of the Process Evaluation framework.*

	Description	Process evaluation questions	Data sources
Context	*Contextual factors that affect implementation, intervention, mechanism of impact and outcomes*	What were the barriers and facilitators to implementing the intervention?	Focus group interviews with staff at 6-month follow-up. Self-reported checklists at 2-month and 6-month follow-up.
Implementation	*Implementation process—What is delivered and how is delivery accomplished?*	To what extent were all modules in the SDM program (including the support tool) implemented?	Self-reported checklists at 2 and 6-month follow-up.
		To what extent did staff from different services participate?	Self-report checklist after staff education
		What procedures were followed to recruit staff for SDM training?	Self-report checklist after staff education
		What adaptations were made to fit the intervention to the context?	Self-report checklists at 2 and 6-month follow-ups.
Mechanisms of impact	*Participants’ response to and interactions with the interventions*	Did staff appreciate *the DST*? Were the staff satisfied with the implementation intervention?	Focus group interviews with staff at 6-month follow-up. Questionnaires to staff^3^ at baseline and 6-month follow-up.

### Data collection and data analysis

The blueprint of components of the Process Evaluation framework (Table III) was used to determine which data sources should be collected for the process evaluation. A considerable amount of data were collected through self-reported checklists, questionnaires and focus group interviews in order to map the conditions for implementation of SDM. As such, both quantitative and qualitative data were collected before, during and after the implementation program. The self-reported checklist was developed by the research team and included 10 items on aspects concerning context, fidelity, dose delivered and reach. This checklist was filled in by the researchers immediately after conducting the brief introduction, the 1-day education and at the 2 and 6-month follow-ups. The checklist was used to capture staff compliance with the content of the education. The results from the checklists were then summarized and qualitatively analysed by the research group, at the same time as the interview data.

At the staff education day, as well as 6 months after, staff were asked to answer two validated questionnaires regarding their knowledge and attitudes to recovery and SDM. The Recovery Knowledge Inventory (RKI) is a 20-item self-reported instrument (Bedregal, O’connell, & Davidson, 2006) that measures the staff’s beliefs and attitudes about recovery among people with mental illnesses. The Dyadic OPTION is a 12-item self-reported instrument (Melbourne, Sinclair, Durand, Légaré, & Elwyn, 2010) that measures perceived patient involvement in SDM from a provider perspective. The data from the questionnaires were analysed with descriptive quantitative analysis using SPSS software 20.0, but due to an unacceptably low-response rate at follow up, these data are not included and reported in this article.

In order to gain greater in-depth knowledge and understanding of the experiences of the staff regarding the implementation activities and the use of the DST, as well as to map barriers and facilitators for implementation, focus group interviews were conducted with a subset of participating staff. All staff that had participated in the education day were invited to participate in one of the interviews. The focus group interviews were conducted at the 6-month meeting following the staff education. A semi-structured interview guide was used, with questions on the extent of use of the DST, usability and impact on care planning and decision making. A total of four focus group interviews were conducted. Three of the six units included in the study belonged to the same psychiatric service and were interviewed together. In total, 29 members of staff, 20 women and 9 men, participated in the interviews which lasted between 15 and 45 minutes. There were between 4 and 15 participants in each group. The focus group interviews were conducted by one of the authors and recorded. In addition, field notes were taken during, and immediately after the interviews, in order to capture the interviewer’s perceptions and reflections during the encounters. These were used to provide context and support the interpretation of the transcripts (Fossey, Harvey, McDermott, & Davidson, 2002).

All focus group interviews were transcribed verbatim and analysed with a qualitative content analysis (Elo & Kyngäs, 2008).Transcriptions were first read inductively multiple times. Three of the authors then began to analyse the data separately. Preliminary codes were defined by highlighting wordings that appeared to capture key thoughts or concepts (Patton, 2002). Seven major codes emerged in the inductive content analysis of group interview transcriptions: (1) Perceptions of limited added value of the DST, (2) Limited embrace of SDM, (3) Barriers associated with service users, (4) Barriers associated with workflow, (5) Intervention-related problems (6) Perceptions of added value with the DST. In the next step, a directed approach followed (Patton, 2002), which involved placing the inductively derived codes into categories based on the process evaluation components from Moore et al. (2015). The idea of employing a deductive method that is derived from an existing model, is that a well-validated model based on a perspective relevant for the study may serve to clarify the results. Findings were discussed within the research team to refine and clarify emerging categories within the data until a consensus was achieved.

Ethical clearance was granted by the Research Ethics Committee at Uppsala University, Sweden (Dnr 2015:218). All participants, both staff and users, gave oral consent to participate after being informed about the study, both verbally and in writing.

## Results

The results that follow are organized according to the components of the process evaluation framework. While as described above, the checklists and focus group interviews were analysed together, the quotes presented below come from the 29 focus group participants (pp. 1–29). The overall outcome of the clinical intervention, that derived from the follow-up interviews and data from the checklists, suggest that the staff perceived the *DST* as user-friendly and useful in supporting participation in different decision-making processes. However, the extent of the use of SDM and the DST was low in all units.

### Contextual factors

Some of the staff stated that the content of SDM was already familiar to them, that working from a participatory perspective was natural in their daily work. These staff described the contents of SDM as *“naturally occurring in the treatment of psychosis*” (p. 16) in order to involve and engage users in their own care. Even if they did not use the steps in the SDM method, they felt that they were following the idea and approach through other methods, such as MI (motivational interviewing), care planning, in therapy and in case management. However, they described their work with user participation as more *“informal and not as systematic, focused and concrete”* (p. 1) as if they had used the decision tool.

Staff suggested that a challenging aspect would be finding the time to introduce users to the DST and to support them in using it. In their experience, it was always difficult to prioritize time for implementing new methods, although once something is put into practice it tends to run smoothly. Their work situation was described as involving having to constantly deal with emergency situations which often become an obstacle for implementation. Even if the notion of encouraging users to write down their experiences and questions beforehand appealed to many of the staff, many felt that finding the time to read these comments would be difficult. One participant described the time challenge in the following manner *“When will I have time to read what the patients have written? It feels like that you don’t really have time to sit with this before you see the patient”* (p. 18). Applying SDM through the DST was perceived as something “over and above” their regular work load and something that required extra time in already stressful situations and in understaffed services. The following quote illustrates the participant’s experience:

“*There is so much, all of the time. There are a thousand things and constant crisis … We always have patients in acute crisis and that makes it very hard to focus”* (p. 16).

Staff at all six sites also expressed a lack of confidence in the willingness and ability of the users to participate in decision-making processes and this reservation affected their readiness to embrace the intervention. Their confidence in the users’ ability to integrate information was low. One participant stated:

*“Sometimes we have to decide … Sometimes a patient has had the opportunity to make their own decision, but then it has not worked out the way they had hoped, and then you just have to take over some decisions”* (p. 2).

Another participant described the mistrust in the user´s ability as *“Like, It’s you (the provider) who are supposed to know about these things. You are best at this”* (p. 3). They described “silent users”, who they considered as unable to express what they want or to take an active part in the decision-making process *“Some patients really do not want to make decisions but want someone else to do it for them”* (p. 16). The staff proposed that the DST was probably more suitable for users with fewer cognitive challenges. Staff described many users as not having the cognitive ability or computer literacy necessary to use the web-based support tool *“I can say that none of my users would be able to follow this on a computer”* (p. 28). Some staff thought that the DST would be most appreciated and useful for younger users who generally have a high degree of digital literacy and who are accustomed to using smart phones in their daily lives.

An additional contextual barrier was the lack of a common organizational agenda supporting a commitment to concretely increasing user participation. User participation and SDM were considered voluntary activity in accordance with staff judgment, experience and attitude. An identified, facilitating factor for implementation was staff agreement that SDM was appropriate for specific formal decisions, such as care planning, assessments and planning at admission and discharge from a service. One participant stated

*“Many times they don’t think they can be involved in decisions made by different authorities, but there really are some things that they do have the chance to influence. A decision support tool like this could help. Before doctor’s appointments or other meetings. That you (the service user) have prepared yourself before such meetings … and then you have gained more power over your own life. Just that you have prepared yourself and can speak your mind and express your opinions” (p. 2).*

Another obstacle concerned the staff´s perception of their own decision-making mandate. Staff sometimes felt that they themselves did not have formal power regarding treatment planning decisions. They described their role as that of providing support to the user in situations where decisions are made in deliberation with for example, doctors or social workers. Some staff felt that the intervention was therefore not appropriate to their service, but would suit services in which more formal decisions were made. The following quote illustrates the participants experiences;

*“The users I meet don’t feel that any decisions are dealt with in our contacts but that decisions relate to bigger things in contacts with social workers and doctors and concern things like assistance and medication. Things that we (case managers) don’t have any influence over” (p. 27).*

The lack of access to digital documentation was also outlined as a contextual barrier. The current movement towards making user journals accessible online had not yet reached the units involved in the implementation process. Information and preferences from the users, recorded in the decision support tool, were therefore not automatically integrated in the regular documentation at the units. As a result, the DST was perceived as an “add-on” to the regular documentation at the units. Staff felt that the DST overlapped with other systems, for example those connected to care planning and crisis plans. One participant described this as follows; “*We write care plans and crisis plans for the service users and there are quite a few things which overlap here, and that can lead to a lot of duplication of effort” (p. 18)*. At the same time, some staff also pointed out that there were questions in the decision tool, directed towards the documentation of user goals and preferences, expectations regarding alternative options, and specifications regarding follow-up, that were appreciated and missed in the regular documentation systems.

### Implementation issues

When studying the process of implementation, it is important to identify both how the effects of the specific intervention occurred and how these effects might be replicated (or avoided) in future interventions of a similar nature. In analysing the follow-up staff interviews, the mechanisms of impact related to contextual factors as well as to factors linked to the implementation intervention itself. One of the components in the intervention, local facilitators, was not developed at any of the sites as planned. The managers of each unit were instructed by the research team to appoint 1-2 local facilitators who would then attend the 1-day training and even receive additional training and supervision from the researchers during the project. Despite the importance of recruiting local facilitators was underlined, none had been appointed. A facilitator was initially appointed at two of the sites, but was subsequently reassigned, and not replaced. Due to the lack of local facilitators, unit managers took on the responsibility of acting as contacts and responsible support persons. Since the unit managers had demanding workloads and lacked knowledge regarding implementation processes, the researchers were forced to step in as external facilitators. While the managers at the participating sites underestimated the need for facilitating the implementation, we as researchers overestimated the managers’ understanding of and ability to recruit facilitators at each site. At the same time, no specific duties or job description for the facilitator had been developed by the research team, except that the facilitator was expected to play an active role in supporting the participating staff, teams and organizations.

An additional contextual factor, related to the difficulties in developing the facilitator role, was the lack of a developed collaboration with users with their own experience of mental illness, who regularly contributed to the development of psychiatric services as consultants, for example, from a user organization. This meant that even in a project focused on developing user influence and participation, users had not been included in the planning and were therefore unavailable during the implementation process. In hindsight, we would have engaged and trained facilitators prior to the start of the process evaluation and developed a clear job description approved by the participating sites.

The information meeting was held in conjunction with an ordinary workplace meeting approximately three weeks before the first training. A brief introduction to the SDM research project and the concept of SDM was included and a written report describing SDM was handed out. The intention was to meet with all staff, and the majority of participants were present during the initial information meeting. The modules making up the 1-day education of SDM were delivered to participating staff. Recorded observations following the seminar suggested that initially, several staff had a different conception of user participation than that which is outlined in SDM models. Some staff felt that they already practiced SDM, using examples which might be described as either simply offering information to users or as noting users’ presence in care planning situations.

The clinical intervention, including the interactive decision tool, was then presented in the education session to the full group by the trainers and a fictive case involving a decision between user and staff was introduced to illustrate the use of the decision tool. Staff were asked to test their skills in completing an SDM process, using a paper version of the DST, since there were not enough computers or pads for all participants in the classrooms. The training was carried out using two specific cases where staff were assigned roles as users as well as staff. In the role play, there was a tendency for staff to get stuck on specific details of the case, and become sidetracked in discussions related to their real life cases rather than practicing the different steps in SDM. The important focus in the skills training in SDM consisted of thoroughly working through each item in the support tool. Direction and supervision from the trainers was therefore needed during the role play. Observations from all six units reinforced that staff were actively involved in the training and contributed to the discussion. The education was appreciated by the participants, with them actively asking questions related to their own work. The training was seen by staff as relevant and understandable and ended with a discussion on the contextual barriers and facilitators for implementing SDM in the services. However, some staff expressed a need for additional implementation support, including one-on-one guidance and supervision at the unit

“*It’s about getting the hang of it. And we haven’t had this hands-on, concrete on-site support and follow up. There has to be someone who is very engaged and has the overall responsibility, who is here, on-site” (p. 16)*.

During the education, the staff expressed their support for SDM and believed that the digital decision tool could help in capturing users’ preferences. Some expressed “relief” at being able to structure their work based on user preferences. The general perception was that SDM, and the use of the digital decision tool, could improve the quality of care, increase user participation, and decrease dissatisfaction among users and their relatives.

*One participant described it in the following way: “Before doctor’s appointments people are nervous, so it’s good to prepare yourself. Like, ‘what do you want to discuss?’ What is important to you?” What issues do you want to raise? Otherwise it becomes a one-way communication, which it most often is. The person is really afraid and doesn’t dare to say anything. And that might be perceived as unwillingness to be involved. It is good to prepare, to write things down” (p. 28).*

However at the same time, a few staff raised concerns about the user’s ability to participate, wondering if it was too difficult for them to use the decision tool, and whether the availability of alternatives would be too stressful for the users. However, the introduction of the decision tool seemed to facilitate consensus around the SDM concepts by concretizing how users could actually be involved. *“Even if you have this way of thinking in your work, it becomes so much more concrete with a structure like this (the DST)” (p. 27).*

### Mechanisms of impact

The staff described the DST as facilitating their ability to work with SDM and as raising their awareness regarding their attitudes when attempting to increasingly involve users in decisions. Despite this positive report, only a minority of staff included in the study had in fact used the DST decision tool in practice. They described their usual practice as demanding that they continuously need to adapt and develop options *“based on what the situation requires”* (p. 2) and expressed that *“in a way we already work like this, but I think I have become more aware thanks to this (the DST) (p. 19)*. The staff that used the DST decision tool with their users, expressed appreciation, especially in regard to the features in the DST which they thought complemented their standard tools. They also felt that using the decision tool together with the users, contributed to their own ability to concretely and actively listen to and consider the users´ opinions and priorities rather than moving too quickly to solutions, something that they were often prone to in stressed situations, and in their eagerness to help. One participant described that:

“*For me this is a confirmation (points at the DST)… To acknowledge the service user. That we, as staff, are reminded to really listen to our service users instead of just handing out a lot of good advice” (p. 16)*.

These staff also described how the DST contributed to increasing their skills and becoming more aware of their own communication patterns: “*I have become more aware and do not pose so many leading questions. I give the users the chance to reflect for themselves and find things out” (p. 18).*

The staff that had used the DST described their users as having appreciated the work with the tool. They also confirmed the fact that users appreciated being able to complete it at home without stress and described the advantages of having the opportunity to go back at any time and review what previously had been written. One participant described it in the following way: “*The fact that they can write down something when they think of it. That it is accessible” (p. 26)*. Another participant highlighted that

“*It can be easier for them to explain how they think when they write it down. And that you summarize and can go back and look at it and revise. So that it becomes a process” (p. 22).*

The staff stressed that DST promotes the importance of being transparent, not selective and discriminative, when sharing information with the users. Although some information may be perceived as uncomfortable, it is still important that it is conveyed and that staff present an accurate and fair view of options and possible consequences. These staff members appreciated how SDM challenges traditional practices where staff say “how it is,” and how SDM can support users in legitimizing their knowledge and increasing their decision-making ability. They also reported that users, who had been introduced to the DST, expressed that it might motivate them to take a more active part in decision-making processes. One participant expressed this in the following way:

*“My experience is that users do not feel that they really have power over their own life. And when I have introduced this (the DST) it´s like something lights up in their eyes. I think it is good that you come with this kind of tool to address this together, to kind of lift this issue of participation” (p. 26).*

Directly after the education, the staff was motivated and committed, but when it came time to implement the intervention in their daily work they fell back into everyday routines since routine work took up all their time. One participant stated

“*Directly after the education you feel very inspired. Then you come back to your ordinary routine with loads of things you have to do… I think we need regular follow-ups. What have you done? How is it going?” (p. 16)*.

An important lesson from the process evaluation is that there would seem to be a gap between ideas and practice in general, but also between initial enthusiasm following the training and everyday routine. A continuous and structured discussion related to employing a new method was requested “*There hasn’t been an active, everyday discussion … And that makes it hard to evaluate how much this method could contribute. Instead of working according to old habits” (p. 1)*. Staff suggested that time and support needed to be allocated for group meetings, where they could discuss the method in relation to their ongoing practice.

They expressed a desire to practice with the DST more, prior to introducing it to the users, in order to feel more confident with the method. They felt that it was difficult to motivate users to use the decision tool if they themselves did not feel familiar with it. One participant described this as follows: *“You want to feel comfortable with it before you introduce it to service users. Otherwise it will be difficult to convince them*…” (p. 29).

## Discussion

To our knowledge, this is the first study employing a process evaluation approach to investigate aspects of a shared decision-making intervention for users with mental illness in psychiatric services in Sweden. This process evaluation address a number of critical issues such as; (1) contextual factors related to the decision making power of both staff and users, to the decision-making ability of users (as assessed by staff) and to the readiness of the service to increase influence and participation, (2) the facilitation role and the need for systematic on-site implementation supervision and support as well as adaptations to the local organization and structures and (3) the potential for integrating research evidence into practice, in this case user participation into routine care.

### Organizational readiness and commitment

Even if staff expressed a general commitment and a positive attitude towards the method, disruptive elements such as organizational changes, personnel turnover and a work flow characterized by constant interruptions related to acute demands, led to a gradual loss of focus on the process. As in all implementation of new methods, manager’s attitudes and level of commitment is crucial (Aarons et al., 2016; Gifford et al., 2013; Moore et al., 2015). Managers need to provide sustainable structures, with a clear vision of the new method as constituting a formal part of the ongoing activities. Although SDM is mentioned in key policy documents in Sweden and internationally, there are no incentives built into organisational structures and it is not promoted systematically at national, regional, or organisational levels (Stovell et al., 2016). A need to foster cultural change among managers, clinicians and users may be seen as well as a prerequisite for structural change, and this is described in the area of user involvement as a considerable challenge (Slade, 2017). The results of the study reinforce the need for a focus on the connection between culture and structure.

In our particular intervention, it became evident that a certain degree of technical readiness in the services, such as access to computers for both staff and users, is an aspect of implementation which should be focused on to a greater degree in the future. Accessibility to computers during the training session would have likely facilitated the interest in and readiness to utilize the tool together with users. Another important aspect of the organizational context was the structure for decision-making and planning in the particular organization. This included both an understanding of the decision hierarchy at the particular service, requirements and expectations for formal decision processes such as treatment planning, and even an understanding of documentation systems which were seen as both a current obstacle and a future possibility for integrating the interactive decision aid in the formal systems regarding user care planning. It also became clear from the results that the SDM process was perceived of as time-consuming by staff, a reaction which suggested that the method should be reserved for decisions which are considered priorities for both the organization and the user. As a result, the next phase of the project will focus on an integration of the decision support tool with the legislated responsibility to create coordinated care plans for users with complex needs, plans that specifically direct the services to develop these plans based on preferences and goals expressed by users.

### The need for facilitation

The need to actively facilitate the implementation process was underestimated and therefore insufficient at all of the participating services. At the same time, staff clearly expressed a need for greater support during the implementation process, with regard to both guidance in the SDM model and support with the concrete use of the DST. The literature clearly reports that facilitators play a key role in supporting staff in understanding what and how they need to change in order to translate evidence into practice (der Zijpp et al., 2016; Eriksson et al., 2016; Harvey & Kitson, 2015). Additionally, a facilitator is more than a role that can simply be assigned or expected. Sanctioned local facilitators would reflect a readiness for change and development, a commitment on the part of the local organization to dedicating resources, a support to the adaptation of interventions to the actual local conditions, and a resource for both technical support (in this case related to DST) and to the attitude change that the intervention may entail (in this case a developing view of participation and capacity among staff). Facilitators with their own “lived experience” of mental health problems and service use might also be seen as a particularly important factor in facilitating the attitude changes the intervention requires so that this change might be seen as connected to other recovery-oriented interventions (Ramon et al., 2017; Slade, 2017). Managing the facilitator role requires however, training and continuous support, a need that clearly was underestimated by the research team. The process evaluation results also suggest that organizational readiness, in this case the contacts with user associations and user advocates that might promote recruiting facilitators, can significantly contribute to this aspect of implementation.

### Perspectives on participation

While there was general agreement among staff regarding the value of user involvement, actual examples of participation by users were difficult to find. The expressed consensus regarding this ideologically described principle, was in other words difficult to define in practice. This finding mirrors international implementation studies on SDM which illustrate that the desire for participation is still greater than the degree of actual participation in practice. In existing research, a number of barriers are outlined related to a successful implementation of SDM, including time constraints, excessive workload of staff, lack of training to staff and users, and lack of access to medical information and decision support to users (Duncan et al., 2010; Morant et al., 2015; Stovell et al., 2016). In the actual implementation, the first training day was initially conceived of as an attempt to lift theories and principles related to participation, to a level that could be discussed and that would lay the groundwork for a shared approach in relation to values, knowledge and required structural changes. During these introductory seminars, staff agreed with the theories and reasoning related to participation, user influence and the recovery process. Others experienced this aspect of the training as too basic, suggesting that user involvement in decision-making and planning was an already integrated part of their practice. One barrier for implementation was therefore what might be referred to as the “we-already-do-that-syndrome,” a somewhat common experience in implementing recovery-oriented interventions (Joseph-Williams et al., 2017). Rather than exploring specific attitudes, assumptions and practice implications, the intervention is dismissed with this reasoning, prior to its implementation. SDM requires both structural change, in terms of health care pathways and delivery, and culture and attitudinal change among clinicians and users (Stovell et al., 2016). The process evaluation, however, revealed additional “hidden” attitudes among staff that may contribute knowledge regarding the challenge of implementing recovery-oriented services such as SDM. Staff, who were very vocal in supporting user participation, nonetheless expressed concerns during the role play, that exposing users to various alternatives might lead to unnecessary stress, and would therefore instead decide which alternatives were most appropriate “for them.” This result, which was elicited in the concrete application of the DST, further supports the idea there is a complex interaction between attitudes and practice that demands further exploration (Joseph-Williams et al., 2017; Slade, 2017).

In order for staff to grasp the difference between informing users and actually sharing power, it may be therefore that working more extensively in a “hands-on” situation with the DST would be required. In the staff training, DST seemed to function as a concrete application for experiencing and learning participation values. For example, in a case where a young man had been called to a meeting due to his insistence on ending the use of medication, staff accepted the individual´s wish to instead discuss his interest in working and earning money (as documented in the decision aid). Staff reported, after working through the decision process step by step, that their attention to and respect for the user´s priorities had actually helped to finally resolve the medication issue, as the user now reported feeling heard. The structure (web-based interactive design) and praxis (learning by doing) came to be seen as key elements of the learning process, and the actual mechanism by which staff could understand and apply the method.

### Methodological considerations

Some methodological reflections should be considered when interpreting the findings. The framework for process evaluation from Moore et al. (2015) was applied to support the planning and designing of the project. The framework has been beneficial for the design of the study and the data collection strategy. However, the implementation has required significant adjustments from the manner in which the original strategy was formulated. The reasons for adjustments were that the services did not comply in many instances with the conditions outlined in the initial strategy. The poor compliance within the services underscores the need for a clearly anchored planning with the services.

Six units were included and 95 staff in these services participated in the intervention. The included units were part of different organizations and with varied functions. This may have impacted the results since municipal social services do not make formal decisions to the same extent as those made in outpatient psychiatry. However, the included services reflect the complexity of today’s psychiatric health care system in Sweden. Social psychiatry, based in the municipal social services, are often challenged by a psychiatric medical model that is dominant in the psychiatric services. Despite this, the municipal social services thought that the intervention, specifically the educational aspect of the intervention, was relevant for them.

### Conclusion and implication for practice

This study emphasizes that implementation of SDM in real-world practice requires a new way of working as staff, and where practice builds on new conceptions of knowledge. User knowledge and skills become central ingredients in the informed decisions that must be made in psychiatric services. The staff however, continue to describe user involvement in terms that reflect attitudes related to promoting compliance in treatment, rather than shared ownership of goals and plans. Organizational priorities, often focused on crisis and acute needs, also distract staff from the often stated vision of a more person centred care. Implementing SDM and user involvement in psychiatric services is clearly work that occurs in the daily meeting between staff and users, but in order to develop this partnership, this direction needs to be a prioritized as a policy and leadership issue.

The intervention was seen as relevant across the range of services involved in this study, but the potential for using the digital DST would seem to be greater in services where formal decisions are a necessary aspect of the organisational care-giving culture. By utilizing a process evaluation to study specific aspects of the implementation of the decision support tool, we were able to clearly discern a number of contextual factors that effected the impact of the intervention. For example, many of the attitudinal and organizational challenges that would later effect the utilization and experience of the tool in practice were discovered during the training process. The impact of the tool itself on the decision-making process became clear, as reluctance to see SDM as a change in practice was mediated by the participation-oriented structure of the tool during the role play. While much attention has been paid to the complex interaction between staff attitudes, organizational cultures and relational aspects of decision-making (Ramon et al., 2017; Slade, 2017), the digital decision support tool included in the intervention here, which builds on interactivity made possible by the web-based tool, suggests that there are concrete aspects of participation that should be focused on in future research.

## Notes

1. Missing n = 3.

2. 3 section managers, 1 project manager, 1 employment coordinator and 2 therapists.

3. Recovery Knowledge Inventory (Bedregal et al., 2006), and Dyadic Option (Melbourne et al., 2010).
